# Mortality Report for the Month of May

**Published:** 1867-07

**Authors:** 


### Mortality Report for the Month of May:—

CAUSES OF DEATH.

| | | | |
|---|---|---|---|
| Abscess, | 1 | Epilepsy, | 1 |
| Accidents, | 11 | Erysipelas, | 1 |
| Apoplexy, | 2 | Fever, Typhoid, | 5 |
| Croup, | 3 | Fever, Lungs, | 6 |
| Cancer, | 3 | Fever, Scarlet, | 5 |
| Consumption, | 32 | Fever, Ship, | 1 |
| Cholera Morbus, | 1 | Fever, Typhus, | 2 |
| Convulsions, | 39 | Fever, Gall, | 1 |
| Canker Sore Mouth, | 1 | Fever, Nervous, | 1 |
| Congestive Chills, | 2 | Fever, Puerperal, | 1 |
| Congestion of Brain, | 2 | Fever, Congestive, | 1 |
| Congestion of Bowels, | 2 | Inflammation of Lungs, | 10 |
| Congestion of Lungs, | 2 | Inflammation of Brain, | 1 |
| Congestion, | 1 | Inflammation of Heart, | 1 |
| Childbirth, | 1 | Measles, | 3 |
| Colic, | 1 | Nephritis, | 1 |
| Diphtheria, | 7 | Old Age, | 5 |
| Drowned, | 6 | Phthisis, | 2 |
| Disease of Heart, | 5 | Poisoning, | 1 |
| Disease of Lungs, | 3 | Pneumonia, | 1 |
| Disease of Brain, | 1 | Small-Pox, | 3 |
| Disease of Liver, | 1 | Stillborn, | 5 |
| Debility, | 3 | Spasms, | 2 |
| Dropsy, | 7 | Suicide, | 1 |
| Diabetes, | 2 | Teething, | 5 |
| Dysentery, | 2 | Whooping-Cough, | 11 |
| Decline, | 1 | Unknown, | 21 |
| Delirium Tremens, | 1 | | |

| | |
|---|---|
| Total, | 241 |
| Total number last year for the month of May, | 275 |
| Total number during the month of April, | 278 |
| Total number during the month of May, | 241 |
| Decrease, | 37 |

DIVISIONS OF THE CITY.

North,......... 56 | South,......... 83 | West,..........111 | Total,..........240

Unknown, 1

Ages of the Deceased.—Under 5 years, 108; over 5 and under 10 years, 9; over 10 and under 20, 9; over 20 and under 28, 35; over 30 and under 40, 30; over 40 and under 50, 17; over 50 and under 60, 13; over 60 and under 70, 12; over 70 and under 80, 5; over 80 and under 90, 3; over 90 and under 100, 1; unknown, 6. Total, 241.

| | | | | | |
|---|---|---|---|---|---|
| Chicago, | 105 | France, | 1 | Sweden, | 6 |
| Austria, | 1 | Germany, | 25 | Scotland, | 2 |
| United States, | 40 | Ireland, | 40 | Unknown, | 2 |
| Canada, | 4 | Isle of Man, | 1 | | |
| England, | 7 | Norway, | 4 | Total, | 241 |

